# RETRACTION: Effect of Transconjunctival Sutureless Vitrectomy Versus 20‐G Vitrectomy on Surgical Wound Cosure in Patients: A Meta‐Analysis

**DOI:** 10.1111/iwj.70357

**Published:** 2025-03-17

## Abstract

**RETRACTION**: HuangY. , SunJ. , WangJ. , ZhangX. , ChenZ. , “Effect of Transconjunctival Sutureless Vitrectomy Versus 20‐G Vitrectomy on Surgical Wound Cosure in Patients: A Meta‐Analysis,” International Wound Journal 21, no. 2 (2024): e14561, 10.1111/iwj.14561.PMC1083410242052905

The above article, published online on 01 February 2024, in Wiley Online Library (http://onlinelibrary.wiley.com/), has been retracted by agreement between the journal Editor in Chief, Professor Keith Harding; and John Wiley & Sons Ltd. Following an investigation by the publisher, all parties have concluded that this article was accepted solely on the basis of a compromised peer review process. The editors have therefore decided to retract the article. The authors did not respond to our notice regarding the retraction.

**RETRACTION**: Y. Huang , J. Sun , J. Wang , X. Zhang , Z. Chen , “Effect of Transconjunctival Sutureless Vitrectomy Versus 20‐G Vitrectomy on Surgical Wound Cosure in Patients: A Meta‐Analysis,” International Wound Journal 21, no. 2 (2024): e14561, 10.1111/iwj.14561.PMC1083410242052905

The above article, published online on 01 February 2024, in Wiley Online Library (http://onlinelibrary.wiley.com/), has been retracted by agreement between the journal Editor in Chief, Professor Keith Harding; and John Wiley & Sons Ltd. Following an investigation by the publisher, all parties have concluded that this article was accepted solely on the basis of a compromised peer review process. The editors have therefore decided to retract the article. The authors did not respond to our notice regarding the retraction.

